# SANA-Biome: A Protocol for a Cross-Sectional Study on Oral Health, Diet, and the Oral Microbiome in Romania

**DOI:** 10.3390/healthcare13172133

**Published:** 2025-08-27

**Authors:** Sterling L. Wright, Oana Slusanschi, Ana Cristina Giura, Ioanina Părlătescu, Cristian Funieru, Samantha M. Gaidula, Nicole E. Moore, Laura S. Weyrich

**Affiliations:** 1 College of Health Solutions, Arizona State University, Phoenix, AZ 85004, USA; 2 Center for Health Through Microbiomes, The Biodesign Institute, Arizona State University, Tempe, AZ 85281, USA; 3 Department of Anthropology, The Pennsylvania State University, University Park, PA 16802, USA; sgaidula@geisinger.edu (S.M.G.); nem5326@psu.edu (N.E.M.); lsw132@psu.edu (L.S.W.); 4 Division of Preventive Dentistry, Faculty of Dentistry, “Carol Davila” University of Medicine and Pharmacy, 050474 Bucharest, Romania; oana.slusanschi@umfcd.ro (O.S.); ana.giura@umfcd.ro (A.C.G.); cristian.funieru@umfcd.ro (C.F.); 5 Department of Oral Pathology, Faculty of Dentistry, “Carol Davila” University of Medicine and Pharmacy, 050474 Bucharest, Romania; ioanina.parlatescu@umfcd.ro; 6 Huck Institutes of Life Sciences, The Pennsylvania State University, University Park, PA 16802, USA; 7 School of Biological Sciences, University of Adelaide, Adelaide, SA 5000, Australia

**Keywords:** oral health, oral microbiome, periodontal disease, Romania, metagenomics, nutrition, epidemiology

## Abstract

Periodontal disease is a widespread chronic condition linked to systemic illnesses such as cardiovascular disease, diabetes, and adverse pregnancy outcomes. Despite its global burden, population-specific studies on its risk factors remain limited, particularly in Central and Eastern Europe. The SANA-biome Project is a cross-sectional, community-based study designed to investigate the biological and social determinants of periodontal disease in Romania, a country with disproportionately high oral disease rates and minimal microbiome data. This protocol will integrate metagenomic, proteomic, and metabolomic data of the oral microbiome from saliva and dental calculus samples with detailed sociodemographic and lifestyle data collected through a structured 44-question survey. This study is grounded in two complementary frameworks: the IMPEDE model, which conceptualizes inflammation as both a driver and a consequence of microbial dysbiosis, and Ecosocial Theory, which situates disease within social and structural contexts. Our aims are as follows: (1) to identify lifestyle and behavioral predictors of periodontal disease; (2) to characterize the oral microbiome in individuals with and without periodontal disease; and (3) to evaluate the predictive value of combined microbial and sociodemographic features using statistical and machine learning approaches. Power calculations based on pilot data indicate a target enrollment of 120 participants. This integrative approach will help disentangle the complex interplay between microbiological and structural determinants of periodontal disease and inform culturally relevant prevention strategies. By focusing on an underrepresented population, this work contributes to a more equitable and interdisciplinary model of oral health research and supports the development of future precision public health interventions.

## 1. Introduction

Periodontal disease is a multifactorial inflammatory condition that affects the supporting structures of the teeth, including the gingiva, periodontal ligament, and alveolar bone [1,2,3]. While its onset is often initiated by microbial biofilms along the gingival margin, its progression is shaped by the complex interplay of biological, behavioral, and environmental factors [3,4,5,6]. Although traditionally regarded as a localized oral health issue, periodontal disease is now widely recognized for its systemic implications. A growing body of evidence links periodontal disease status to a range of chronic diseases, including Alzheimer’s disease [7,8], type 2 diabetes [9,10,11,12,13,14], cardiovascular disease [9,15,16,17,18], and adverse pregnancy outcomes [19,20,21], underscoring the critical role of oral health in overall systemic well-being.

Our understanding of periodontal disease has advanced by studying the oral microbiome, a diverse community composed of not only bacteria [22,23,24] but also archaea [25], fungi [25,26], viruses [27,28,29], and protozoa [30,31]. These microbial entities interact dynamically with each other and with the host, modulating local and systemic immune responses [32,33]. However, the high interindividual variability, temporal fluctuations, and diverse ecological niches (e.g., dental plaque and saliva) within the oral cavity complicate efforts to define a universal “healthy” oral microbiome [34,35,36].

While considerable progress has been made in identifying specific microbial taxa associated with periodontal disease because of advances in “omic” technologies [37,38,39,40], a single microbiome-based signature has not emerged [41,42]. Moreover, the social and biological determinants that initiate and sustain periodontal disease remain poorly understood in a single population, let alone on a global scale. To comprehensively understand the etiology of periodontal disease, it is essential to move beyond strict biological frameworks and incorporate social determinants of health, including diet, hygiene practices, lifestyle factors, and access to dental care. It is critical that we also include diverse sample types (i.e., saliva, plaque, and calculus) and work across diverse populations that may maintain distinct oral microbiota due to their distinct genetics, life histories, diets, and environmental exposures [43,44,45]. This integrative perspective is particularly important in high-risk populations, where structural and behavioral factors may interact with microbial dynamics in unique and synergistic ways.

In Romania, where oral diseases are particularly high, there is a notable lack of up-to-date epidemiological data on periodontal health [46]. This scarcity of data hinders the development of targeted prevention and treatment strategies and limits our understanding of how social and environmental conditions specific to a culture shape oral health outcomes. Addressing periodontal disease in Romania is therefore essential not only for improving oral health but also for advancing overall population health and reducing health disparities.

We developed the SANA-biome Project to identify biological and social determinants of periodontal disease. The acronym SANA is symbolically inspired by the Romanian words “Sănătate orală” (oral health), Alimentație (diet), Natură (environment), and Antropologie (anthropology). This study aims to study and identify key predictors of periodontal disease within a cross-sectional Romanian cohort by integrating oral microbiome data with in-depth sociodemographic, lifestyle, and health surveys. By integrating saliva and dental calculus samples with participant questionnaires on oral hygiene, systemic health, diet, and socioeconomic factors, our goal is to better characterize both biological and social risk factors for periodontal disease in Romania, a country with disproportionately high rates of oral disease [47,48] and limited microbiome research [49]. For instance, a World Health Organization study reported that over 70% of Romanian children experience dental caries, a rate markedly higher than that observed in many Western European countries [50].

## 2. Methods/Study Design

### 2.1. Theoretical Framework

To guide the interpretation of our findings, we ground our study in two frameworks. First, we draw on the Inflammation-Mediated Polymicrobial-Emergence and Dysbiotic-Exacerbation (IMPEDE) model. This model, developed by Van Dyke et al. (2020) [51], offers a contemporary understanding of the ecological and inflammatory processes that underlie periodontal disease. It builds upon previous hypotheses [52,53] by emphasizing that periodontitis is not driven by specific microbial taxa but rather by a community-wide shift in the oral microbial community that triggers and sustains a host inflammatory responses. Specifically, the IMPEDE model hypothesizes that periodontal disease begins with an inflammatory stimulus, which may be environmental or behavioral in origin. This inflammation creates an environment that is selective for the emergence of pathobionts that are normally suppressed in a healthy state. These emerging microbes form a synergistic, polymicrobial community that is functionally distinct from the health-associated microbiota. As these microbes proliferate, they further stimulate the host immune system, intensifying inflammation and resulting in a self-sustaining cycle of microbial imbalance and tissue destruction. Thus, in this model, inflammation is both a driver and a consequence of microbial dysbiosis.

To complement the IMPEDE model, we will also apply Ecosocial Theory, developed by Krieger [54,55], to contextualize disease risk within broader social, political, and ecological forces. Central to Ecosocial Theory is the concept of embodiment—the idea that social experiences, from access to healthcare to food security, become biologically embedded and influence health outcomes. This theory emphasizes the importance of structural conditions, such as socioeconomic status, education, gender, and geography, as fundamental causes of health inequities. In line with this theory, our study incorporates a comprehensive questionnaire designed to access not only oral hygienic behaviors but also the social determinants of health that shape periodontal disease risk in Romania. This is important as this population has higher risk for oral diseases compared to other countries in the European Union [47,48,56,57]. By capturing information on diet, dental hygiene, income, education, healthcare access, stress, and cultural practices, we aim to identify whether these upstream factors are strong predictors for the composition of the oral microbiome. This approach aligns with Ecosocial Theory’s call to analyze pathways to embodiment, accountability, and cumulative exposure over the life course.

Together, the IMPEDE and Ecosocial frameworks position the SANA-biome Project as a biocultural investigation of periodontal disease. It integrates microbial profiling with social context to advance our understanding of how biological and structural forces interact to produce oral health disparities. By bridging molecular data with social science perspectives, this work contributes to a more holistic and equitable version of oral health research.

### 2.2. Aim and Design

This study is designed to investigate whether lifestyle choices and the oral microbiome are associated with periodontal disease in a Romanian cohort. In this study, we aim to accomplish the following:

1. Identify lifestyle and behavioral predictors of periodontal disease in a Romanian cohort. Participants will complete a 44-question survey capturing a wide range of lifestyle, dietary, and oral hygiene behaviors and self-reported perception of oral health status and dental attendance. Participants will also complete the validated Romanian version of the Oral Health Impact Profile (OHIP-14) questionnaire [58,59].
2. Characterize the saliva and dental calculus microbiome of individuals with and without periodontal disease using shotgun metagenomic and amplicon sequencing, proteomic, and metabolomic strategies. This analysis will generate high-resolution taxonomic and functional potential profiles, enabling comparisons of alpha diversity, beta diversity, and microbial function, as well as phylogenetic analysis.
3. Integrate social and microbiome data to develop predictive models of periodontal disease risk. We will evaluate how lifestyle, demographic, and microbial features interact to improve the prediction and understanding of periodontal disease susceptibility in Romanian individuals.

### 2.3. Rationale for Studying Saliva and Dental Calculus in the Context of Periodontal Health

Saliva and dental calculus are two complementary substrates that offer distinct advantages for investigating the oral microbiome and its relationship to periodontal health. Saliva represents a dynamic, non-invasive sample type that reflects the composite microbial community and immune activity from across the oral cavity and, to some extent, the systemic environment [60,61,62]. Its includes microbes, host-derived proteins, metabolites, and signaling molecules, many of which have been linked to the onset and progression of periodontal disease [62,63,64].

In contrast, dental calculus is the calcified form of dental plaque and serves as a long-term, stable substrate of the oral microbiome [65,66,67]. Once plaque mineralizes into calculus, it preserves DNA, proteins, and metabolites within a hardened matrix, protecting biomolecules from rapid degradation [68]. This stability enables researchers to examine a more representative snapshot of oral health [67,69]. Both subgingival and supragingival calculus have been associated with diet, hygiene, salivary flow, and periodontal disease [65,70,71].

This study specifically focuses on supragingival calculus, which harbors microbial taxa and functional pathways relevant to periodontal health. Unlike subgingival calculus, it allows for comparable analysis while minimizing certain methodological drawbacks [72]. For instance, subgingival calculus communities can be influenced by probing depth, introducing variability that is difficult to control for in systematic analyses [73]. Moreover, subgingival microbial communities are more likely to be impacted by other confounding effects, such as gingival bleeding [74,75]. By targeting supragingival calculus, we are also able to directly compare samples from individuals without periodontal disease, enhancing the interpretability of the findings.

### 2.4. Ethical Considerations

The study protocol was approved by the Research Ethics Committee from the “Carol Davila” University of Medicine and Pharmacy (PO-35-F-03, 2022), Bucharest, Romania. The study protocol and data collection documents were also reviewed by the Arizona State University Institutional Review Board, and it was determined that the protocol was exempt pursuant to Federal Regulations 45CFR46 (#IRB 00021824) on 9 May 2025.

Participants are given a written description of the project, its purpose, and the procedure. The investigators are also available to further clarify any questions. Patients who agree to participate and sign the informed consent form will be included in this study.

Participants may withdraw from this study at any time. The information documents contain details regarding this aspect. The samples and data related to them will be discarded.

Confidentiality is ensured throughout this study so that the participants’ personal data will not be explicitly linked to their biological samples or to their responses to the questionnaire. To achieve this, each participant in this study will be assigned a code that will be inscribed on the sample containers, on the questionnaire, and on the charts. Only the investigator that performs the sampling and dental examination will know the identity of the participant.

### 2.5. Participants and Procedures

#### 2.5.1. Subject Eligibility and Recruitment

Participants will be eligible for inclusion in this study based on the following criteria.

Inclusion criteria are as follows:

1. Individuals must be patients who are already scheduled for a professional oral prophylaxis appointment at a dental clinic, independent of this study.
2. Participants must be between 20 and 50 years of age.
3. Participants must be fluent in either Romanian or English.
4. For participants with periodontal disease, individuals must have a probing pocket depth greater than 3 mm.

Exclusion criteria are as follows:

1. Individuals younger than 20 or older than 50 years of age.
2. Individuals who have taken antibiotics within the past 3 months.
3. Individuals with dentures, fewer than three remaining teeth, or no mandibular teeth.
4. Individuals who are currently pregnant or breastfeeding.
5. Individuals diagnosed with malignancies or undergoing chemotherapy or on immunosuppressive treatment.

Study participants will be recruited through two pathways. The primary approach involves selecting eligible patients who present for dental services at the UMF “Carol Davila” Clinics. Collaborating clinicians will identify patients who meet the inclusion criteria and express interest in this study and refer them to the principal investigators (PIs) at UMF “Carol Davila” for consultation and sample collection. All referred individuals will be screened for eligibility prior to enrollment. This collaboration is important not only to ensure an adequate participant enrollment but also to provide a representative sample of patients from diverse social backgrounds and various areas across capital city.

Patients receiving care at the dental clinics who are identified as potential candidates for this study during their initial dental consultation will be invited to participate in this study. Investigators and collaborating clinicians will provide preliminary information about this study and distribute the informed consent and participation agreement documents. Upon signing, participants will complete the lifestyle questionnaire, followed by a dental examination and biological sample collection, including dental calculus and saliva.

Approximately 5 mL of saliva will be collected first, followed by the collection of supragingival dental calculus. Both samples will be placed in labeled tubes containing ZYMO DNA/RNA Shield and stored at −20 °C. Each tube will be marked with a unique, de-identified participant code to ensure confidentiality. Samples will be temporarily stored at the collection site and later transported to Arizona State University in insulated containers with cold packs to maintain temperature stability during transit and preserve sample integrity for downstream multiomic analysis.

#### 2.5.2. Assessment of Oral Health Status

Following the oral examination, dental status will be recorded using a modified version of the WHO oral health assessment form for adults [76]. This adaptation allows for comprehensive recording of clinical attachment level (CAL) for all teeth rather than limiting measurements to the six index teeth as outlines in the original WHO form.

Recorded variables will include dental health indicators such as the presence of cavities, erosion, and existing restorations, as well as periodontal health markers including bleeding on probing (BoP), periodontal pocket depth (PPD), and CAL. Periodontal assessments will be performed using a WHO periodontal probe by two calibrated examiners. Each tooth will be examined at six sites (mesio-buccal, buccal, disto-buccal, mesio-lingual, lingual, and disto-lingual), and the highest PPD and CAL values per tooth will be recorded.

Periodontal disease will be diagnosed using the 2018 classification system, defined as interdental CAL ≥3 mm at two or more non-adjacent teeth [77]. Participants with PPD or CAL values below 3 mm and BoP greater than 10% will be classified as having gingivitis, while those with PPD and CAL values below 3 mm and BoP ≤ 10% will be categorized as having no periodontal disease. Severity of periodontal disease will be determined in accordance with the New Classification from the 2017 World Workshop on Periodontal and Peri-Implant Disease and Conditions [78] (Table 1).

The oral examination performed in this study will follow standard clinical protocols.

#### 2.5.3. Covariates Included in the Questionnaire

The questionnaire captures an extensive range of covariates aimed at contextualizing oral microbiome profiles through behavioral, sociodemographic, and health-related factors. Participants are first asked about general background information, including nationality, current and past places of residence (classified as rural or urban), length of time spent in each location, education level, and main sources of drinking water. Data on dental attendance frequency and perceived access to care are also collected.

A core section of the questionnaire is devoted to dietary habits, including detailed questions on the frequency of consumption of various food and drink categories such as fresh fruits and vegetables, sweets, tea, coffee, meat, dairy, and fermented products. Participants are also asked about tobacco and alcohol use.

General health questions include current medications, systemic illnesses, recent infections, and the use of antibiotics or anti-inflammatory drugs. Oral health is assessed through self-reported perceptions of dental and gingival health, frequency of dental visits, number of natural teeth, presence of dentures, and oral hygiene practices. The latter includes brushing frequency, tools used (e.g., floss, interdental brushes), and use of specific products like toothpaste and mouthwash. The questionnaire also includes items from the Oral Health Impact Profile (OHIP) which assesses the psychosocial and functional impacts of oral health.

Importantly, this instrument addresses a critical gap in the field: the absence of a standardized metadata framework for capturing social, dietary, and behavioral information in oral microbiome studies [79,80,81]. This limitation has hampered cross-study comparisons and meta-analyses. By systematically collecting these data, this study contributes to establishing a more comprehensive and reproducible standard that can enhance future integrative analyses in oral health research.

### 2.6. Questionnaire Analysis

As part of our first hypothesis, we will conduct a comprehensive analysis of the questionnaire data to evaluate how individual lifestyle factors are associated with periodontal disease. This analysis will focus on behavioral and sociodemographic variables collected via the questionnaire.

The questionnaire captures a broad range of self-reported exposures, including the following:

- Oral hygiene practices (e.g., frequency of toothbrushing, flossing, mouthwash use, dental visit history).
- Dietary intake patterns (e.g., frequency of consuming processed foods, added sugars, fresh fruits and vegetables, and fermented foods).
- Sociodemographic and environmental variables (e.g., age, education, income level, access to dental care, household size).

We will use multivariable logistic regression models to assess the association between these variables and periodontal disease status. Where appropriate, categorical variables (e.g., use of toothbrush) will be treated as ordinal predictors to preserve meaningful gradation. Continuous predictors will be centered and scaled to facilitate model convergence and interpretability.

To further refine our model, we will apply stepwise regression, LASSO regularization, and Akaike Information Criterion (AIC)-based model comparison to identify the most predictive features. This analysis will allow us to

1. Identify independent lifestyle predictors of periodontal disease;
2. Detect broader trends in health behaviors that may disproportionately affect high-risk individuals;
3. Inform feature selection for inclusion in our downstream multiomic integration models.

### 2.7. Sample Size Calculation

Calculating statistical power in microbiome studies remains a challenge due to the inherent uncertainty in estimating true effect sizes between microbial community structure and variables of interest. To determine a reasonable sample size for detecting group-level differences in oral microbiome composition, we conducted a multivariate power analysis using the Evident plugin in QIIME2 (v.2024.10) [82].

**Figure 1:**
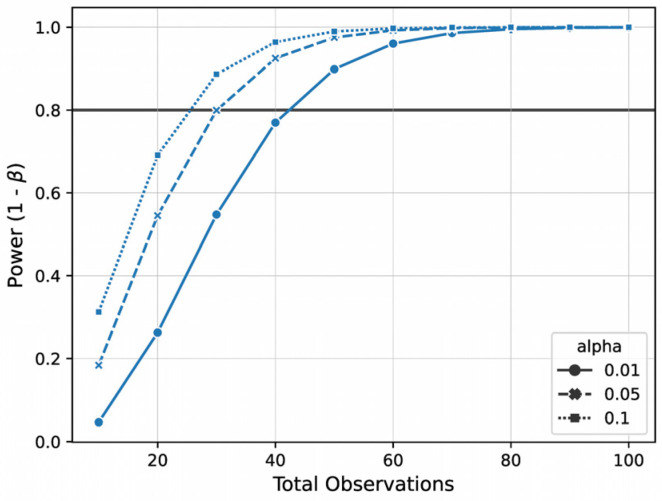
Power analysis results based on Aitchison distance with periodontal disease status as the grouping variable (*n* = 22). Data was generated with amplicon sequencing strategy.

We conducted a post hoc power analysis on an Aitchison distance matrix derived from species-level saliva microbiome pilot data (*n* = 22) and used periodontal disease status as the grouping variable. Power estimates were generated across a range of total sample sizes (from 10 to 100) and at three commonly used significance thresholds (α = 0.01, 0.05, and 0.10) (Table 2). As shown in Figure 1, the power curve indicates that achieving 80% (1 − β) requires a minimum of approximately 60 total observations (i.e., ~30 individuals per group) to achieve a significance level of α = 0.05. At α = 0.01, at least 80 observations will be necessary to achieve comparable power.

Our power analysis indicated a sample size of 120 participants would be sufficient to detect significant effects at a significance level of α = 0.05, assuming a medium effect size (Cohen’s f = 0.693). However, we acknowledge that this sample size may limit our ability to fully adjust for multiple potential confounders, such as sex, age, and BMI, and may not provide sufficient power to detect smaller effect sizes or complex interaction effects. In particular, associations involving more nuanced dietary variables (e.g., frequency of consuming fermented foods) may exhibit weaker or more context-specific effects that are harder to detect in a study of this size. As such, while this study is adequately powered for key drivers of periodontal disease, the inability to fully model the influence of all potentially relevant variables should be considered a limitation.

### 2.8. Laboratory Protocols

#### 2.8.1. Oral Microbiome Analysis

DNA will be extracted from 500 μL of saliva using a MagMAX Microbiome Ultra Nucleic Acid Isolation kit (Applied Biosystems, Waltham, MA, USA) as per the manufacturer’s instructions with minor modifications, eluting in 50 μL of elution solution. Extraction blank controls (EBCs) will be included at the start and end of each extraction to monitor laboratory contamination. DNA extracts will be quantified following extraction using a Qubit dsDNA HS Assay (Invitrogen, Carlsbad, CA, USA) to confirm DNA presence and extraction success.

DNA sequencing libraires will be prepared by either amplifying the V4 hypervariable region of the 16S rRNA gene using the 515F 806RB primer set, as previously described [83], with Platinum Taq DNA Polymerase, High Fidelity (Invitrogen), or by conducting shotgun metagenomic sequencing [84]. The thermocycling conditions will be set as follows: 6 min denaturation at 95 °C followed by 38 cycles of 95 °C for 30 s, 50 °C for 30 s, and 72 °C for 90 s and a final extension of 60 °C for 10 min [85]. A no-template control will be included in the amplification process to monitor contamination downstream of the extraction [86]. All the EBCs will be included in the amplification as well. Following amplification, all samples and controls will be quantified using a Qubit dsDNA BR Assay (Invitrogen), pooled in equal molarity, and purified at 1.1x using an AxyPrep Mag PCR clean-up kit (Axygen, Union City, CA, USA). The final 16S amplicon sample pool will be quantified using a Tapestation and D1000 reagents (Agilent, Santa Clara, CA, USA) and Qubit dsDNA BR Assay (Invitrogen, Carlsbad, CA, USA).

#### 2.8.2. Host Proteome Analysis

Proteomic characterization will be performed using a SomaScan^®^ assay (SomaLogic, Inc., Boulder, CO, USA), a highly multiplexed, aptamer-based proteomic platform that quantifies thousands of human proteins across a wide dynamic range [87,88]. The assay utilizes SOMAmer^®^ (Slow Offrate Modified Aptamer) reagents to bind proteins with high specificity and affinity. For this study, we will use the 11K SomaScan panel, which measures approximately 11,000 protein targets.

In brief, we will follow the protocols described in a previous study [89]. Human saliva samples (~5 mL per sample) will be incubated with SOMAmers, which are tagged with a fluorophore, biotin, and a photocleavable linker. Protein–aptamer complexes are captured on streptavidin-coated beads, washed to remove unbound material, and subjected to UV light to release the complexes. Following further washing and a competitive binding step to reduce non-specific interactions, the SOMAmers are re-captured and then released from the proteins for quantification. Data will undergo SomaLogic’s standard normalization pipeline, including hybridization control normalization, median signal normalization on calibrator samples, plate-scale normalization, and inter-plate calibration to minimize technical variability.

For quality assurance, replicate samples and calibrator controls will be included across plates. All analyses will be conducted using fully normalized data, as defined in SomaLogic’s bioinformatics guidelines. This normalization approach has been shown to reduce the median inter-plate coefficient of variation.

#### 2.8.3. Saliva Metabolomic Analysis

Untargeted LC-MS metabolomics will be employed to identify metabolite signatures associated with periodontal disease and lifestyle factors such as smoking and diet. Approximately 50 μL of each sample will be placed in a 2 mL Eppendorf vial. The initial step for protein precipitation and metabolite extraction will be performed by adding 500 μL MeOH and 50 μL internal standard solution (containing 1810.5 μM ^13^C_3_-lactate and 142 μM ^13^C_5_-glutamic acid). The mixture will then be vortexed for 10 s and stored at −20 °C for 30 min, followed by centrifugation at 14,000 RPM for 10 min at 4 °C. The supernatants (450 μL) will be collected into a new Eppendorf vial and dried using a CentriVap Concentrator (Labconco, Fort Scott, KS, USA). The dried samples will be reconstituted in 150 μL of 40% PBS/60% ACN. A pooled sample will be a mixture of all saliva samples and will be used as the quality-control (QC) sample.

Protocols for the untargeted LC-MS metabolomics method have previously been published [90]. Briefly, all LC-MS experiments will be performed on a Thermo Vanquish UPLC-Exploris 240 Orbitrap MS instrument (Waltham, MA, USA). Each sample will be injected twice, 10 µL for analysis using negative ionization mode and 4 µL for analysis using positive ionization mode. Both chromatographic separations will be performed in hydrophilic interaction chromatography (HILIC) mode on a Waters XBridge BEH Amide column (150 × 2.1 mm, 2.5 µm particle size, Waters Corporation, Milford, MA, USA). Using a mass spectrometer equipped with an electrospray ionization (ESI) source, we will collect untargeted data from 70 to 1050 *m*/*z*. To identify peaks from the MS spectra, we will make extensive use of in-house chemical standards (~600 aqueous metabolites). In addition, we will search the resulting MS spectra against the HMDB library, Lipidmap database, METLIN database, as well as commercial databases including mzCloud, Metabolika, and ChemSpider. We will use Thermo Compound Discoverer 3.3 software for aqueous metabolomics data processing. The untargeted data will be processed for peak picking, alignment, and normalization.

### 2.9. Data Processing and Analysis

#### 2.9.1. Microbiome Data Processing and Analysis

Sequencing data will be assessed using FastQC (v0.11.9), and adapters will be trimmed using Trimmomatic (v0.39) [91]. To preserve participant privacy, human-associated reads will be removed using KneadData (v0.10.0) by aligning against the GRCh38.p14 human reference genome. Low-complexity reads will be filtered using Komplexity [92], and duplicate reads will be removed with Seqkit [93].

Taxonomic profiling will be conducted with Kraken2 (v2.1.2) [94], and species-level abundance estimates will be refined using Bracken (v2.7). Functional profiling will be performed using HUMAnN3 (v3.6) [95], which generates normalized pathway abundance and coverage tables for each sample, enabling comparative functional analysis.

Downstream statistical analyses will be performed using QIIME2 [96,97] and R [98]. Alpha diversity will be assessed using the following metrics: observed features richness, Faith’s phylogenetic diversity, and the Simpson index. Beta diversity will be measured using Aitchison distance, unweighted Jaccard, and UniFrac distances to evaluate between-sample compositional differences. Ordination will be performed using Principal Coordinates Analysis (PCoA), and group-level differences in beta diversity will be tested using PERMANOVA [99].

To identify microbial features associated with periodontal disease status, we will first conduct differential abundance testing using MaAsLin3 (Microbiome Multivariable Associations with Linear Models) [100]. In our model, periodontal status (presence or absence of disease) will be included as the primary fixed effect. Additional covariates will be included based on results from the multivariable logistic regression models and AIC-based model comparisons. This two-step approach ensures that only the most predictive and parsimonious set of covariates is incorporated into the MaAsLin3 models. To assess site-specific microbial associations with periodontal disease status, separate analyses will be conducted for saliva and supragingival calculus.

To further explore shifts in microbial community structure, we will perform differential connectivity (DC) analysis using the SOHPIE, a R program to facilitate a differential network analysis of finding differentially connected taxa [101]. This approach constructs group-specific microbial association networks using SparCC and calculates degree centrality for each taxon. A robust pseudo-value regression model will then be used to identify taxa whose centrality values significantly differ between periodontal health and disease while adjusting for the same covariates identified through AIC-based selection.

#### 2.9.2. Multiomic Integration and Analysis

After identifying key microbial taxa and functional pathways associated with disease status, we will integrate these microbiome features with host proteomic and metabolomic profiles to investigate coordinated host–microbe interactions.

To explore whether the salivary microbiome, proteome, and metabolome, as well as the dental calculus microbiome, jointly contribute to periodontal disease status, we will apply IntegratedLearner, a Bayesian ensemble framework developed for robust, interpretable multiomics prediction and classification [102]. IntegratedLearner enables the integration of multiple omics layers alongside associated metadata to construct probabilistically grounded models of disease prediction.

Within this framework, Bayesian Additive Regression Trees (BARTs) will serve as the base learners for each data layer (microbiome, proteome, metadata), and predictions from each layer will be combined in a meta-learning stage using either non-negative least squares or rank loss minimization. This ensemble approach captures both nonlinearities and interaction effects across datasets and provides posterior credible intervals, offering a measure of uncertainty around both predictive performance and biomarker importance.

Although this study uses a cross-sectional design, IntegratedLearner is particularly well-suited for evaluating whether integrating data from multiple biological layers improves the classification of periodontal disease status compared to single-omic models. By incorporating this framework, we will identify multiomic signatures that distinguish individuals with periodontal disease from those without and assess how host–microbe interactions and lifestyle factors jointly contribute to disease susceptibility. This integrative approach supports the development of more accurate, data-driven models for oral health risk stratification and contributes to a deeper understanding of the systems-level biology underlying periodontal disease.

### 2.10. Data Sharing and Availability

This study is committed to open science and adheres to the FAIR data principles (Findable, Accessible, Interoperable, and Reusable). Upon completion of this study and publication of results, all relevant datasets and supporting materials will be made publicly available through appropriate repositories. Questionnaire responses and participant metadata will be shared as Supplementary Materials with future manuscripts and deposited in the National Microbiome Data Collaborative (NMDC) Portal, which is designed to support standardized, interoperable metadata for multiomic studies [80]. Metagenomic sequencing data will be submitted to the NCBI Sequence Read Archive (SRA) under a dedicated BioProject accession. Proteomic data will be uploaded to MassIVE (Mass Spectrometry Interactive Virtual Environment) [103], and metabolomic data will be deposited in the Metabolomics Workbench [104]. All code and analytical pipelines used for data processing and statistical analysis will be made available on GitHub, with accompanying documentation to facilitate transparency and reproducibility. Data and code will be released under open licenses when possible to support reuse by the broader research community. Additional details can be found in our STORMS checklist, which is provided in the Supplemental Materials.

The questionnaire and STORMS checklist can be found on Zenodo: https://doi.org/10.5281/zenodo.16757905.

## 3. Discussion

Periodontal disease remains one of the most prevalent chronic conditions worldwide, with significant implications for oral and systemic health. Despite its high burden, population-specific microbiome data—particularly in Central and Eastern Europe—remains limited [49]. This study represents a novel effort to address this gap through community-based research design that integrates saliva and dental calculus collection with participant-reported data on oral hygiene, diet, systemic health, and socioeconomic factors. By triangulating microbial data from two distinct oral niches with rich contextual information, this protocol provides a multidimensional framework to explore the biological and social risk factors contributing to periodontal disease.

At present, participant enrollment is ongoing. Our pilot study using 16S rRNA amplicon sequencing on an initial subset of saliva samples has been completed. These pilot data informed our power calculations and helped refine both the sampling strategy and our planned multiomic integration framework. The insights gained have guided the development of sequent analyses incorporating shotgun metagenomic sequencing, salivary proteomics, and untargeted metabolomics.

Dental calculus offers a valuable archive of long-term oral microbial exposure and is uniquely suited to reflect chronic inflammation and biofilm maturation processes associated with periodontal pathology [32,36,37,52,71]. When analyzed alongside salivary microbiomes, which capture more dynamic and transient changes in the oral cavity [43,105,106,107], these data can reveal both rapid and enduring microbial patterns. The addition of a detailed questionnaire enables the exploration of how behavioral and structural factors shape these microbiomes over shorter and longer time spans, offering insights into the lived experiences that may contribute to disease risk.

The strengths of this approach lie in the integration of biomolecular and self-reported data to assess periodontal risk from both biological and sociocultural perspectives. By combining oral microbiome sequencing from multiple niches (saliva and dental calculus) with detailed lifestyle, dietary, and health questionnaires, this study offers a multidimensional view of the factors contributing to oral disease. This approach allows for the identification of both microbial signatures and upstream behavioral and structural determinants, advancing beyond reductionist models that isolate biology from context. In addition to metagenomic sequencing, this study also adopts a multiomic strategy, incorporating metabolomic and proteomic data. This layered approach enables a more comprehensive understanding of microbial function, host response, and metabolic interactions, providing a systems-level perspective on periodontal disease. The inclusion of validated survey instruments (e.g., OHIP-14) alongside high-resolution molecular data further enhances the reproducibility and comparability of findings across studies.

This design is especially important in Romania, where high rates of periodontal disease intersect with a lack of up-to-date epidemiological data and persistent underrepresentation in global oral microbiome research [49]. By focusing on this population, our study not only addresses a significant regional data gap but also contributes to broader questions about how health disparities, cultural practices, and systemic inequities shape oral health outcomes.

Despite these strengths, our study also has a few limitations that should be acknowledged. First, because we collect a wide range of behavioral and sociodemographic variables, we acknowledge that it will not be possible to fully control for all potential covariates. Given our planned sample size and power calculations, our analyses will be best suited to detect medium or large effect sizes and may be underpowered to identify more subtle associations. Additionally, we are not collecting clinical biomarkers such as serum inflammatory markers, triglyceride levels, or other systemic indicators that are commonly used in clinical settings. This limits our ability to triangulate oral health outcomes with systemic health measures.

Another key limitation is the cross-sectional nature of our study design. Without additional follow-ups, we are unable to capture intraindividual variation over time or make inferences about the directionality or causality of observed associations between the oral microbiome, behavior, and periodontal disease. Moreover, we cannot infer the relative risk. Nevertheless, the identified associations can inform hypotheses and predictive modeling for future longitudinal studies.

Furthermore, much of the data on lifestyle, diet, and oral hygiene is self-reported. Although self-reported data may be subject to recall bias or social desirability bias, prior studies have demonstrated that self-reported dietary habits and responses to instruments like the OHIP-14 are meaningfully correlated with clinical indicators of oral disease severity [47,48,59,76,108]. Thus, while these limitations are important to consider, our integrative and community-focused approach still provides valuable insights into the biological and cultural factors contributing to oral health disparities in Romania.

In future analyses, statistical modeling will incorporate specification curves and model selection methods (e.g., IntegratedLearner [102]) will help assess the robustness and predictive values of various combinations of biological, behavioral, and demographic variables. This will allow us to evaluate which variables, alone or in combination, are most consistently associated with periodontal disease. By testing model performance across a range of analytic choices, we aim to minimize researcher bias and better identify stable, generalizable predictors of periodontal disease risk in Romania.

Ultimately, this study will inform the development of precision oral health interventions and community-tailored prevention strategies in Romanian populations at higher risk for periodontal disease and beyond. It highlights the value of interdisciplinary approaches that integrate biological anthropology, microbiology, epidemiology, and public health to advance our understanding of complex oral diseases.

## 4. Conclusions

The SANA-biome Project represents a critical step toward disentangling the biological and social determinants of periodontal disease in Romania. By integrating saliva and dental calculus microbiome data with detailed dietary, behavioral, and sociodemographic information, this study will generate one of the most comprehensive datasets to date on oral health in Eastern Europe. Grounded in both the IMPEDE model and Ecosocial Theory, our protocol bridges molecular biology and anthropology, providing a biocultural framework for understanding how structural, behavioral, and microbial factors interact to shape disease risk.

Although limited by its cross-sectional design, SANA-biome lays the foundation for longitudinal and intervention-based studies that will further clarify causal pathways and culturally tailored prevention strategies. The combination of multiomic analyses with validated health and lifestyle surveys not only enhances reproducibility but also sets a precedent for standardized metadata collection in oral microbiome research. Importantly, the community-based and interdisciplinary nature of this work ensures that the findings will have relevance not only to scientific audiences but also to dental practitioners, public health officials, and policymakers seeking to reduce the burden of periodontal disease.

Ultimately, this study contributes to a growing body of evidence that oral health cannot be separated from broader social and ecological contexts. By situating the Romanian experience within global conversations on oral health inequities, SANA-biome will inform precision public health interventions and advance a more holistic, culturally grounded understanding of the oral microbiome and its role in systemic health.

## Figures and Tables

**Table 1:** Classification of periodontal disease severity. This table outlines the diagnostic criteria used to determine the severity (Stage I–IV) and extent of periodontitis in study participants. Classification is based on clinical attachment level (CAL), probing pocket depth (PPD), and other clinical and radiographic indicators. These criteria will be used to stratify participants during analysis of microbial and proteomic profiles.

Classification of Periodontal Disease Severity	Diagnostic Criteria
Mild—Stage I	Interdental CAL 1–2 mm Maximum PPD ≤ 4 mm
Moderate—Stage II	Interdental CAL 3–4 mm Maximum PPD ≤ 5 mm
Severe—Stages III and IV	Interdental CAL ≥ 5 mm Maximum PPD ≥ 6 mm Tooth loss due to periodontal disease

**Table 2:** Power analysis results.

Alpha	Total Observations	Power	Effect Size	Metric	Column
0.01	30	0.55	0.693	cohens_f	Periodontal disease
0.05	30	0.800	0.693	cohens_f	Periodontal disease
0.1	30	0.887	0.693	cohens_f	Periodontal disease

## Data Availability

No new data were created.

## Supplementary Materials

The following supporting information can be downloaded at: https://www.mdpi.com/article/10.3390/healthcare13172133/s1. File S1. Participant information sheet. Information sheet given to eligible study participants about this study. Consent will be obtained after the information sheet has been completely read and understood by the participant. File S2. Consent form. This consent form requires a patient’s signature on agreeing to participate in this study. File S3. Questionnaire. The questionnaire is given to participants who provided consent.
