# Supplementary material for: SANA-Biome: A Protocol for a Cross-Sectional Study on Oral Health, Diet, and the Oral Microbiome in Romania
Source: Healthcare (Basel). 2025 Aug 27;13(17):2133. doi: 10.3390/healthcare13172133 (PMC12427674; doi:10.3390/healthcare13172133)
Supplement: Supplementary file 1 [file healthcare-13-02133-s001.zip › File S1. Participant-information-sheet.pdf]

PARTICIPANT INFORMATION FORM

You are being asked to participate in a study conducted by Dr. Oana Slusanchi, Dr. Ioanna Parlatescu, Dr. Laura Weyrich, and Sterling Wright. You are welcome to keep this form for your records. This form provides some background information about our research and helps explain why we are conducting this study.

Why are we doing this project?
Over the past decade, researchers have begun to understand that the bacteria in your mouth play a critical role in your health. By sequencing the bacterial DNA in plaque and saliva, scientists have found that one bacterium cannot determine whether a person will develop periodontal disease. Instead, researchers have found that it is the communities of microbes (such as, bacteria, viruses, fungi) and their interactions that can help predict whether one will develop periodontal disease.

Many things can influence what types of bacteria live in your mouth. Where you live, what you eat and drink, and how often you brush your teeth may all play a role in deciding which bacteria can thrive in your mouth. This is why we had you fill out the questionnaire asking you many questions about your lifestyle. We expect that people who share similar lifestyles will also share similar bacteria.

Your participation is important as it will provide key information for oral health professionals in Romania. Again, the data we collect from your saliva, plaque, and questionnaire will be anonymized but will be used to study how oral bacteria are associated with lifestyle. Similar research is occurring in America and other parts of Europe, but every population is unique. This is why the results from this study are so critical because they may help dental hygienists develop better therapeutic strategies specifically for people living in Romania with periodontal disease. This could mean that one day there will be less people with mouth pain and oral diseases. It could also save people money from having to go to the dentist.

What are we collecting?

We are collecting three types of information: a general assessment of your oral health (for example, if you have periodontal disease or not); a questionnaire about your lifestyle; and oral samples (saliva and dental plaque).

We are collecting a general assessment of your oral health because we are recruiting people to this study who both do and do not have periodontal disease. It is important for us to understand what type and severity of periodontal disease you have to understand if certain microbes are linked to how severe periodontal disease can be.

We are collecting a questionnaire about your lifestyle because we want to understand why certain microbes live in people's mouths. We know that the food you eat, the places you go, and your oral hygiene can impact the types of microbes that we find in people's mouths. However, this have never been examined in Romania, and the factors that shape the microbes in people's mouths may be different in Romania compared to other parts of the world.

We are collecting both saliva and plaque to look at the microbes that are present in your mouth. As of now, it is not entirely clear whether the bacteria in your saliva or plaque can help make better predictions for periodontal disease. We will examine all of the microbes (bacteria, viruses, fungi, etc.)

that live in your saliva and dental plaque by looking at their DNA, or genetic information. We will not examine any human DNA as part of this study.

What do I do if I want to withdraw from the project? Or if I have further questions about the project?

As a reminder, your participation is voluntary. You may contact the researchers of this project anytime (please find our contact information below) about concerns or questions that may arise, or to have your data removed from the study. You will not be penalized for wanting to be withdrawn from the study or pressured to remain in the study.
