# Supplementary material for: SANA-Biome: A Protocol for a Cross-Sectional Study on Oral Health, Diet, and the Oral Microbiome in Romania
Source: Healthcare (Basel). 2025 Aug 27;13(17):2133. doi: 10.3390/healthcare13172133 (PMC12427674; doi:10.3390/healthcare13172133)
Supplement: Supplementary file 1 [file healthcare-13-02133-s001.zip › File S2. ConsentForm.pdf]

PennState

CONSENT TO PARTICIPATE IN THE RESEARCH

Informed Consent

Please read this consent document carefully before you decide to participate in this study. The researcher will answer any questions before you sign this form.

Study Title: The associations between the oral microbiome and lifestyle in one of the most high-risk populations for periodontal disease in Europe

What is this project about?

- To see whether there are associations between different oral health, lifestyles, and the types of bacteria living in the mouth. This is done by identifying the species of bacteria living in plaque or calculus (*i.e.,* hard calcified plaque). We will be looking at the DNA of the bacteria only, no human DNA will be analyzed. We will see if people with periodontal disease have the same microbes in their mouths as people who do not have periodontal disease. We will then use the responses on the questionnaires to see if people with similar diets or lifestyles also share similar oral bacteria.
- Periodontal disease is impacting thousands of people living in Romania. Currently, there are few therapeutic treatments to help prevent and cure it. The results from this study will hopefully provide some insights into how oral health professionals can develop better treatments for periodontal disease.

Who is involved in this project?
This project is being conducted by:
Oana Slusanchi (Carol Davila University)
Ioanina Parlatescu (Carol Davila University)
Laura Weyrich (Penn State University)
Sterling Wright (Penn State University)

Why are we asking you to participate?
We are asking patients like you to participate because your saliva, plaque/calculus, and answers to the questionnaire can be useful to understand the types of microbes that are linked to periodontal disease and why people may develop these microbes.

What will you be asked to do?
You will be asked to sign this consent form recognizing that your participation is voluntary. You will then be asked to complete a questionnaire that will take approximately ~15 minutes to complete. You will then be asked to spit in a tube and allow the dental hygienist to place your plaque or calculus sample into a tube for the study. Northing further is required.

Are there any risks associated with participating in this project?

There are no ain acquiring your plaque, calculus, or saliva, sample. There are also no risks in completing the questionnaire. The procedure in collecting your plaque or calculus will be done the same way even if you were to not enroll in the study.

What are the benefits of this research project?
Understanding how your lifestyle can impact the types of bacteria in your mouth is key for researchers understanding periodontal disease. By answering the questions on the questionnaire and providing plaque/calculus and saliva samples, you will provide us some insight on how and why periodontal disease is so prevalent in Romania. Results from this research may help us find ways to prevent and treat oral diseases in Romania. This means that more people in Romania will have healthier mouths. It could also mean that people like you could save time and money from having to go to the dentist.

Can I withdraw from this project?
Your participating is completely voluntary. If you agree to participate, you can still withdraw from the study at any time. You will be given a participant information with the contact information for the researchers so that you can contact them to remove yourself from the study if you change your mind.

What will happen to your plaque/calculus samples and questionnaire?
All samples and questionnaires for this project will be anonymized. This means your personal details, such as your name, will not be attached to the samples. No one will be able to associate you with the research and any publications. Any remaining sample tissue will be destroyed after ten years. All of the results and data that are created during this study will be published and made publicly available in this anonymous format. This means that anyone in the world will be able to download the anonymized data and information about the microbes produced in this study.

Are you okay with being recontacted for a follow up study? (Circle one) **Y / N**

Agreement:
I have read the procedure described above. I voluntarily agree to participate in the procedure, and I have received a copy of this description.

Participant (Print and Sign): ____ Date:________

Principal Investigator (Print and Sign): ______________________________________ Date:________
