# Supplementary material for: SANA-Biome: A Protocol for a Cross-Sectional Study on Oral Health, Diet, and the Oral Microbiome in Romania
Source: Healthcare (Basel). 2025 Aug 27;13(17):2133. doi: 10.3390/healthcare13172133 (PMC12427674; doi:10.3390/healthcare13172133)
Supplement: Supplementary file 1 [file healthcare-13-02133-s001.zip › File S3. Questionnaire.pdf]

1. Participant ID Number________________________________

2. Date of birth (dd/mm/yyyy)_____________________________

3. Sex: M/F/N ____

4. Date of interview (dd/mm/yyyy)__________________________

5. Interviewer's name___________________________________

Why do we ask these questions?

We are interested in improving health, especially oral health. In this study, we are exploring the microorganisms (such as bacteria, viruses, and other small organisms) that live in your mouth and how this may be related to how you live and your health. In particular, we are investigating how your lifestyle can impact the bacteria in your mouth that can cause perdiodontal disease.

The information that you provide today will be incredibly useful when we try to understand how different lifestyles impact different types of bacteria, and how these signals are linked to oral health. We will also be exploring which bacteria can cause oral disease, such as periodontal disease. Together, this information will help us understand how and why periodontal disease occurs in Romanians.

Everything you say is confidential. We do not collect your name with the answers, so we collect only what has been said, but not who said it.

If you have any questions, please ask. We want you to feel relaxed.

INSTRUCTIONS

The QUESTIONNAIRE

This questionnaire asks about your background, diet, general health, and oral health. We would like you to complete the questionnaire even if you have good dental health.

HOW TO ANSWER THE QUESTIONS

Each question is on the left side of the page and is inside a grey box. Some questions have boxes. You can answer these questions by either shading or marking the box to indicate your answer. Other questions will require you to write in your response. Please right in the designated area to answer these questions.

EXAMPLES

A1. What is your nationality?	Romanian<br>☑	Multiple<br>☐	Other<br>☐
A4. What city are you currently living and how long have you lived there?			
Bucharest_5 years			

A. THESE QUESTIONS ARE ABOUT YOUR BACKGROUND

We are ask these questions about your background because they may provide useful information in understanding your oral health and bacteria.

A1. What is your nationality?	Romanian<br>☐	Multiple<br>☐	Other<br>☐	
A2. What your first language?	Romanian<br>☐	Multiple<br>☐	Other<br>☐	
A3. Usual place of residence	Urban<br>☐	Rural<br>☐	Suburban<br>☐	Not sure<br>☐
A4. What city are you currently living and how long have you lived there?				
______________________________				
A5. If you had lived in other cities, when did you live there and for how long?				

A6. What level of education have you completed?	No formal schooling…………………. ……………………………….☐<br>High schooling completed……….………………………………..☐<br>College/University completed………………..……………………☐<br>Postgraduate degree…………………………………………….……☐

A7. What is your main source of drinking water?	Urban tap water ☐	Rural tap water ☐	Bottled water ☐	Other ☐ Please specify: ______

A8. During the last year, have you avoided or delayed visiting a dentist because of cost?	Yes ☐1	No ☐2	Rather not say ☐3

A9. How much difficulty would you have paying a €100 dental bill?	None ☐	Hardly any ☐	A little ☐	Very difficult ☐

B. THESE QUESTIONS ARE ABOUT YOUR DIET

How often do you consume the following items?

B1. Fresh fruit	Never ☐	Monthly ☐	Weekly ☐	Daily ☐	Did in the past, but have stopped for more than one year ☐
B2. Fresh vegetables	Never ☐	Monthly ☐	Weekly ☐	Daily ☐	Did in the past, but have stopped for more than one year ☐

B3. Store bought sweets/candy	Never ☐	Monthly ☐	Weekly ☐	Daily ☐	Did in the past, but have stopped for more than one year ☐
B4. Home baked sweets	Never ☐	Monthly ☐	Weekly ☐	Daily ☐	Did in the past, but have stopped for more than one year ☐
B5. Soft drinks such as Coca Cola	Never ☐	Monthly ☐	Weekly ☐	Daily ☐	Did in the past, but have stopped for more than one year ☐
B6. Hot Green Tea	Never ☐	Monthly ☐	Weekly ☐	Daily ☐	Did in the past, but have stopped for more than one year ☐
B7. Hot Black Tea	Never ☐	Monthly ☐	Weekly ☐	Daily ☐	Did in the past, but have stopped for more than one year ☐
B8. Coffee	Never ☐	Monthly ☐	Weekly ☐	Daily ☐	Did in the past, but have stopped for more than one year ☐
B9. Meat	Never ☐	Monthly ☐	Weekly ☐	Daily ☐	Did in the past, but have stopped for more than one year ☐
B10. Milk	Never ☐	Monthly ☐	Weekly ☐	Daily ☐	Did in the past, but have stopped for more than one year ☐
B11. Fermented dairy (e.g. yogurt or cheese)	Never ☐	Monthly ☐	Weekly ☐	Daily ☐	Did in the past, but have stopped for more than one year ☐

B11. Fermented foods	Never	Monthly	Weekly	Daily	Did in the past, but have stopped for more than one year
	☐	☐	☐	☐	☐

How often do you use any of the following types of products (Read each item)

B12. Cigarettes or Cigars	Never ☐	Monthly ☐	Weekly ☐	Daily ☐	Did in the past, but have stopped for more than one year ☐
B13. Chewing tobacco	Never ☐	Monthly ☐	Weekly ☐	Daily ☐	Did in the past, but have stopped for more than one year ☐
B14. Electronic cigarettes	Never ☐	Monthly ☐	Weekly ☐	Daily ☐	Did in the past, but have stopped for more than one year ☐
B15.a. Alcohol	Never ☐	Monthly ☐	Weekly ☐	Daily ☐	Did in the past, but have stopped for more than one year ☐
B15.b. What types of alcohol do you drink?<br>List all that apply	______________________________				

C. GENERAL HEALTH

We ask these questions about your general health because your general health influences your microbes.

C1. Are you currently taking medication for anything?	Yes	No	Rather not say
	☐1	☐2	☐3

C2. If so, what medications are you taking.		____________________	
C3. Do you have a current OR have you recovered from a recent infection within the last 3 months? (Not including COVID-19)	Yes<br>☐1	No<br>☐2	Rather not say<br>☐3
C4.a. Have you ever tested positive for COVID-19?	Yes<br>☐1	No<br>☐2	Rather not say<br>☐3
C4.b. If so, how long ago were you tested?	____________________		
C5. Do you currently use any medications to treat inflammation?	Yes<br>☐1	No<br>☐2	Rather not say<br>☐3
C6. Have you taken any antibiotics in the past six months?	Yes<br>☐1	No<br>☐2	Rather not say<br>☐3
C7.a. Have you been diagnosed with any systemic diseases?	Yes<br>☐1	No<br>☐2	Rather not say<br>☐3
C7.b. If so, what is it?	Please specify ____________________		

D. ORAL HEALTH PERCEPTIONS

We are asking these questions because your oral health and the microbes within your mouth are connected.

During the last 12 months have you had (tick all that apply)

D1. Would you rate your oral health as:	Excellent ☐<br>Very good ☐<br>Good ☐<br>Average ☐<br>Poor ☐<br>Very poor ☐

		Teeth	Gums
D2. How would you describe the state of your teeth and gums?	Excellent	☐	☐
	Very good	☐	☐
	Good	☐	☐
	Average	☐	☐
	Poor	☐	☐
	Very poor	☐	☐

	Yes	No	Rather not say
D3. A bad taste in your mouth	☐1	☐2	☐3

	Never	Hardly ever	Occasionally	Fairly often	Very often
D4. How often do your gums bleed?	☐1	☐2	☐3	☐4	☐5

	Very often	Fairly often	Occasionally	Hardly ever	Never
D5. How often do you see a dentist?	☐	☐	☐	☐	☐

	0 natural teeth	1-9 teeth	10-19 teeth	20 or more teeth
D6. How many natural teeth do you have?	☐	☐	☐	

				☐
D7. Do you have any removable dentures?	A partial denture ☐	A full upper denture ☐	A full lower denture ☐	A full upper and lower denture ☐

	Never	Monthly	Weekly	Daily	Did in the past, but have stopped for more than one year
D8. How often do you clean your teeth?	☐	☐	☐	☐	☐

		Yes	No
D9.a. Do you use any of the following to clean your teeth? (Read each item)	Toothbrush…………………	☐	☐
	Wooden toothpicks………..	☐	☐
	Plastic toothpicks……………	☐	☐
	Thread (dental floss) ………..	☐	☐
	Mouthwash………… ………..	☐	☐

D9.b. Other(s) (Please specify)	______________________________

	Yes	No
D9.c. If you use a toothbrush, do you use toothpaste?	☐	☐

	Never	Monthly	Weekly	Daily	Did in the past, but have stopped for more than one year
D9.d. If you use mouth rinse, how often do you use it?	☐	☐	☐	☐	☐

D9.e. What is the name of the mouth rinse you use?	______________________________

D10. Have you had trouble pronouncing any words because of problems with your teeth, mouth, or dentures?	Never ☐	Hardly ever ☐	Occasionally ☐	Fairly often ☐	Very Often ☐	Don't Know ☐
D11. Have you felt that your sense of taste has worsened because of problems with your teeth, mouth, or dentures?	Never ☐	Hardly ever ☐	Occasionally ☐	Fairly often ☐	Very Often ☐	Don't Know ☐
D12. Have you had painful aching in your mouth?	Never ☐	Hardly ever ☐	Occasionally ☐	Fairly often ☐	Very Often ☐	Don't Know ☐
D13. Have you found it uncomfortable to eat any foods because of problems with your teeth, mouth, or dentures?	Never ☐	Hardly ever ☐	Occasionally ☐	Fairly often ☐	Very Often ☐	Don't Know ☐
D14. Have you been self-conscious because of your teeth, mouth, or dentures?	Never ☐	Hardly ever ☐	Occasionally ☐	Fairly often ☐	Very Often ☐	Don't Know ☐
D15. Have you felt tense because of problems with your teeth, mouth, or dentures?	Never ☐	Hardly ever ☐	Occasionally ☐	Fairly often ☐	Very Often ☐	Don't Know ☐

	Never	Hardly ever	Occasionally	Fairly often	Very Often	Don't Know
D16. Has your diet been unsatisfactory because of problems with your teeth, mouth, or dentures?	☐	☐	☐	☐	☐	☐
D17. Have you had to interrupt meals because of problems with your teeth, mouth, or dentures?	☐	☐	☐	☐	☐	☐
D18. Have you found it difficult to relax because of problems with your teeth, mouth, or dentures?	☐	☐	☐	☐	☐	☐
D19. Have you been a bit embarrassed because of problems with your teeth, mouth, or dentures?	☐	☐	☐	☐	☐	☐
D20. Have you been a bit irritable with other people because of problems with your teeth, mouth, or dentures?	☐	☐	☐	☐	☐	☐
D21. Have you had difficulty doing your usual jobs because of problems with your teeth, mouth, or dentures?	☐	☐	☐	☐	☐	☐

D22. Have you felt that life in general was <u>less satisfying</u> because of problems with your teeth, mouth, or dentures?	Never ☐	Hardly ever ☐	Occasionally ☐	Fairly often ☐	Very Often ☐	Don't Know ☐
D23. Have you been <u>totally unable to function</u> because of problems with your teeth, mouth, or dentures?	Never ☐	Hardly ever ☐	Occasionally ☐	Fairly often ☐	Very Often ☐	Don't Know ☐
